# Common lipid features of lethal ventricular tarchyarrhythmias (LVTAs) induced by myocardial infarction and myocardial ion channel diseases

**DOI:** 10.1038/s41598-017-04620-w

**Published:** 2017-06-26

**Authors:** Jiayan Wu, Qian Wu, Dian Wang, Jing Kong, Wentao Dai, Xingxing Wang, Xiaojun Yu

**Affiliations:** 1 Department of Forensic Medicine, Shantou University Medical College, Shantou, 515041 China; 2 Shanghai Center for Bioinformation Technology, Shanghai, 201203 China; 3 2nd Affiliated Hospital, Affiliated Hospital, Shantou University Medical College, Shantou, 515041 China

## Abstract

Lethal ventricular tachyarrhythmia (LVTA) is the most prevalent electrophysiological underpinning of sudden cardiac death (SCD), a condition that occurs in response to multiple pathophysiological abnormalities. The aim of this study was to identify common lipid features of LVTA that were induced by distinct pathophysiological conditions, thereby facilitating the discovery of novel SCD therapeutic targets. Two rat LVTA-SCD models were established to mimic myocardial infarction (MI) and myocardial ion channel diseases. Myocardial and serum specimens were analyzed using ultra-performance liquid chromatography-mass spectrometry (UPLC-MS)-based lipidomics. The lipid profiles of the myocardial and serum specimens were similar between the models. Eleven myocardial lipid classes were altered, including downregulations of: cardiolipin, ceramide, phosphatidylinositol, phosphatidylethanolamine, triacylglycerol, diacylglycerol, phosphatidylglycerol, lysophosphatidylethanolamine and phosphatidylserine, and upregulations of: lysophosphatidylcholine and phosphatidic acid. Serum concentrations of triacylglycerol, lysophosphatidylcholine, phosphatidylethanolamine and phosphatidylinositol were also altered. Alterations of lipids in paired myocardia and sera were closely correlated. Cardiolipin 70:5, cardiolipin 74:9 and ceramide d34:2 were tested as potential biomarkers of LVTA. The results indicate that there are common LVTA lipid profiles induced by MI and myocardial ion channel diseases, potentially offering novel LVTA-SCD therapeutic targets.

## Introduction

Sudden cardiac death (SCD) is rapid, unexpected death that occurs from cardiac dysfunction. SCD remains a major public health problem worldwide, accounting for an estimated 15–20% of all deaths^1^. The most prevalent electrophysiological events leading to SCD in 60–80% of pathological conditions are lethal ventricular tachyarrhythmias (LVTA), particularly ventricular tachycardias (VT) and ventricular fibrillations (VF)^2^. Controlling incidence of LVTA is critical to prevent LVTA-related SCD. Previous studies showed that impaired cardiac metabolisms occurring due to ongoing cardiac diseases induced life-threatening ventricular arrhythmias and cases of SCD^3–6^. Therefore, the elucidation of LVTA metabolism is vital for the identification of novel preventive and therapeutic SCD targets. It is relevant and interesting to determine whether LVTA-SCD events occurring by different pathophysiological mechanisms share metabolic features. A previous study showed that acute myocardial infarction (MI)-induced LVTA shared myocardial metabolic features with LVTA events induced by myocardial ion channel diseases. The metabolic profiles shared the downregulations of five fatty acids^7^. Hence, we hypothesized that LVTA may share lipidomic features, regardless of their pathological origins.

Lipids have an array of biological functions, including: intracellular signaling, energy storage and metabolism, maintaining plasma membrane structural integrity, and antioxidant and mitochondrial respiratory activities^8, 9^. Most of these roles have been correlated with overall myocardial tissue functions and electrophysiological activities. Additionally, lipid disruptions were associated with tachyarrhythmia^6, 7, 10, 11^. However, no study to date has investigated global regulation of lipid species in myocardia of LVTA subjects.

This study aimed to describe lipidome of LVTA caused by either MI or myocardial ion channel diseases, and to determine whether their lipidomes had common features. The study also aimed to screen any commonly deregulated lipid species as potential biomarkers. In previous studies, two rat LVTA-SCD models were developed^7^. The first model induced LVTA-SCD using aconitine (ACO), a C19-diterpenoid alkaloids that induced LVTA by disrupting myocardial ion channel activities^12^. The second model of LVTA-induced SCD was induced by coronary artery ligation (CAL) producing MI events. Using these models, lipidomes were characterized using a non-targeted ultra-performance liquid chromatography-mass spectrometry (UPLC-MS) approach. Common differentially-expressed lipid species were identified in the myocardia of the two independent rat LVTA models. Lipid-related metabolic pathways and correlation networks were analyzed. The abilities of the differential lipids to diagnose LVTA-SCD were assessed. In addition, commonly deregulated lipid species in paired serum samples were screened and used to validate the lipidomes identified in the LVTA myocardia.

## Results

### Echocardiogram and hemodynamic features of two rat LVTA models

**Figure 1:**
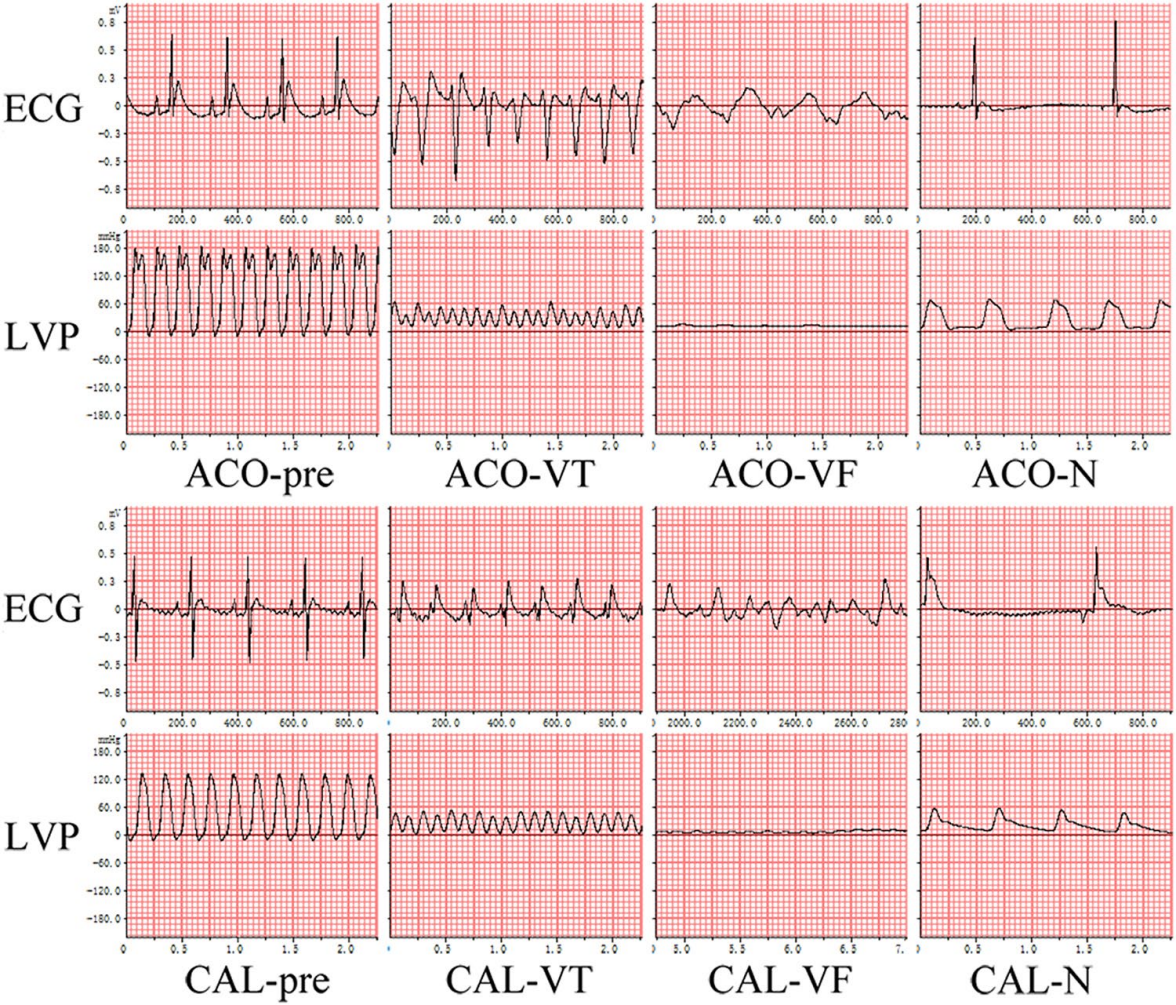
ECG and hemodynamic features of two LVTA models and controls. Both LVTA models (ACO-VT, ACO-VF, CAL-VT, CAL-VF) experienced VT, VF, and a dramatic decrease in LVP. Control subjects (ACO-N and CAL-N) experienced bradycardia and mildly decreased LVP when compared to controls; ACO-pre and CAL-pre: measurements taken prior to treatments; measurements during VT or VF in LVTA models; ECG: electrocardiogram; LVP: left ventricular pressure; VT: ventricular tachycardia; VF: ventricular fibrillation.

Thirteen ACO-LVTA rats and seven ACO-N (control) rats were developed. The mean durations of VT, VF and the arrhythmia scores of the ACO-LVTA rats were 62.4 s, 104.8 s and 5.7, respectively. In ACO-N rats, the durations of VT, VF and the arrhythmia scores were 58.3 s, 23.4 s and 3.0, respectively (*P* < 0.05 for VF and arrhythmia scores when comparing the two groups, Fig. 1 and Table 1).

**Table 1:** Electrophysiological parameters of the experimental models.

	Model I			Model II			AT vs. CT
	AN	AT	AN *vs*. A T	CN	CT	CN *vs*. CT	
VT(s)	58.3 ± 69.9	62.4 ± 32.0	0.862	10.6 ± 18.7	100.5 ± 83.6	0.003	0.156
VF(s)	23.4 ± 40.7	104.8 ± 54.6	0.003	1.5 ± 5.3	157.6 ± 97.1	<0.001	0.120
Score	3.0 ± 2.8	5.7 ± 0.7	0.034	1.6 ± 1.4	6.3 ± 0.7	<0.001	0.058

Notes: AN, AT: the control and LVTA group in Model I, respectively; CN, CT: the control and LVTA group in Model II, respectively; AN vs. A T, CN vs. CT, AT vs. CT: *P*-value calculated by a *t*-test between AN and AT, CN and CT, and between AT and CT, respectively. The data are presented as “mean ± SD”.

Additionally, 12 CAL-LVTA rats and 12 CAL-N (control) rats were developed. The durations of VT, VF and the arrhythmia scores of the CAL-LVTA rats were 100.5 s, 157.6 s and 6.3, respectively. The durations of VT, VF and the arrhythmia scores for the CAL-N rats were 10.6 s, 1.5 s and 1.6, respectively. (*P* < 0.05 for all three parameters, when the two groups were compared, Fig. 1 and Table 1). Durations of VT, VF and the arrhythmia scores were not significantly different between the ACO-LVTA and CAL-LVTA subjects (*P* > 0.05, Table 1).

**Figure 2:**
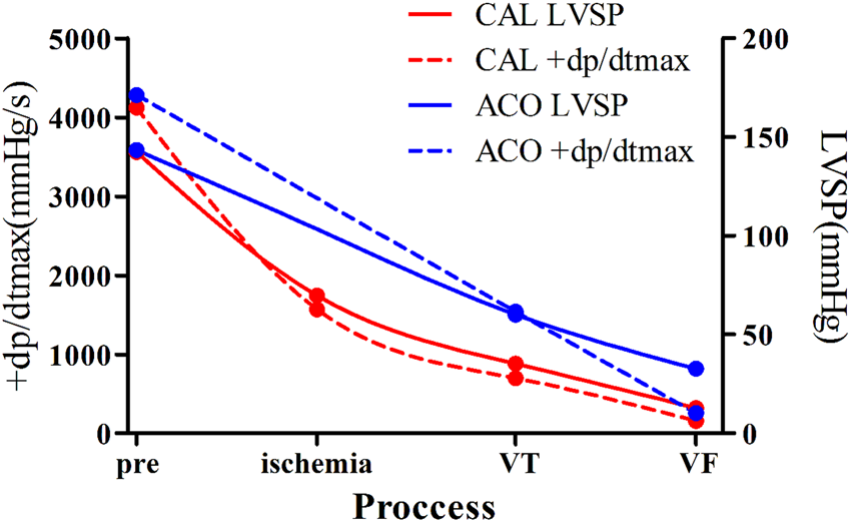
Change tendencies of left ventricular systolic pressures and +dp/dtmax values in LVTA models. The cardiac functions dramatically decreased in both LVTA models in particular after VT/VF, as illustrated by LVSP and +dP/dtmax data graphed in the figure. LVSP: left ventricular systolic pressure; +dP/dtmax: the maximum left ventricular pressure increase; pre: prior to aconitine injection or CAL operation; ischemia, VT and VF represent ligation time points in CAL operations and the onsets of VT and VF, respectively.

During LVTA events, the functions of left ventricles, indicated by left ventricular systolic pressure (LVSP) and maximum left ventricular pressure rise (+dP/dtmax), fell abruptly down to zero in the rats that died of SCD. LVSP changes and maximum left ventricular pressure rise had similar trends between the two LVTA models (Fig. 2).

### Myocardial and serum LVTA lipid profiles

According to the exact masses determined by both positive and negative ion modes, a total of 1,010 myocardial lipids and 746 serum lipids were identified, representing 23 lipid classes. Partial least squares-discriminant analysis (PLS-DA) models were established using unit variance (UV) scaling to provide an overview of the lipid profiles. Apparent separations between the LVTA models and their respective controls were observed both in the myocardia and sera (Supplementary Fig. 1s), indicative of significant differences in lipid levels.

### Myocardial and serum lipidomes were similar in two LVTA models

**Figure 3:**
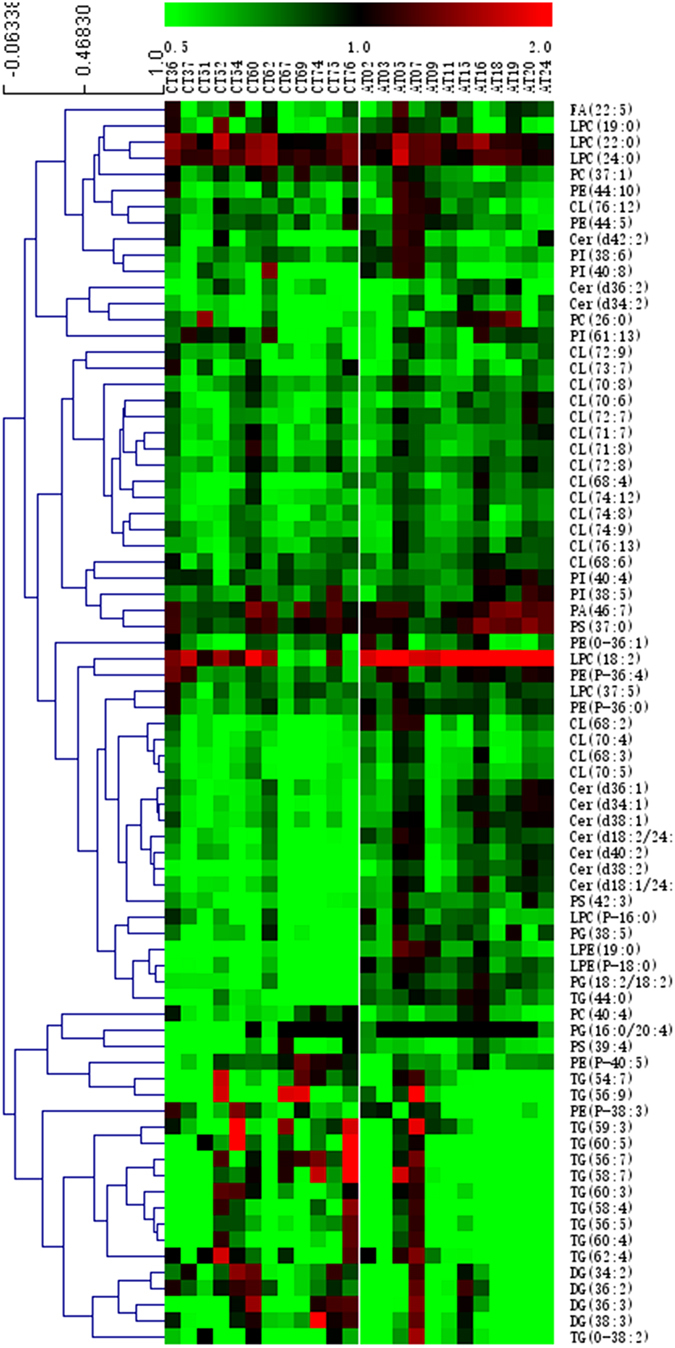
A heatmap of the change tendencies of 77 lipid species that were altered in LVTA myocardial tissues. The colors from green to red in each square indicate the ratio of LVTA-induced differential lipid abundances in each myocardial sample over the average abundances of the corresponding lipids in respective controls. Green represents downregulation in abundance while red represents up-regulation.

In the myocardia, there were 377 and 417 respective lipid species that were altered in the ACO-LVTA and CAL-LVTA models when compared to matched controls [variable important in projection (VIP) > 1.0 or *P* < 0.05)]. Among them, 174 lipids were altered in both models; 77 lipids shared change tendencies between the two LVTA models. Detailed specifications of the 77 lipids are given in Supplementary Table 1. To illustrate the landscape of lipids profiles altered in the LVTA models, a heatmap was constructed of common differentially altered lipids from calculations of the ratios of every LVTA model subject’s lipid abundances to the average control abundances of the same lipid species. Overall, these lipids showed significant abundance differences when comparing the two LVTA groups to their respective controls (Fig. 3).

To validate the data indicating that myocardial lipids were altered by LVTA, serum lipidomes from paired subjects were analyzed with the same lipidomics strategies. Forty-six lipids were commonly altered in the sera of the two LVTA models. However, these species were not the same as those that were commonly altered in the myocardia (Supplementary Table 2). Ideally, the differential lipids in the myocardia would have similar alterations in paired sera, suggesting good validation of the myocardial results. However, such as a validation was not achieved here given the discrepancies between the LVTA myocardial and serum lipidomes. Thus, two other strategies were used to validate the myocardial lipid profiles.

**Figure 4:**
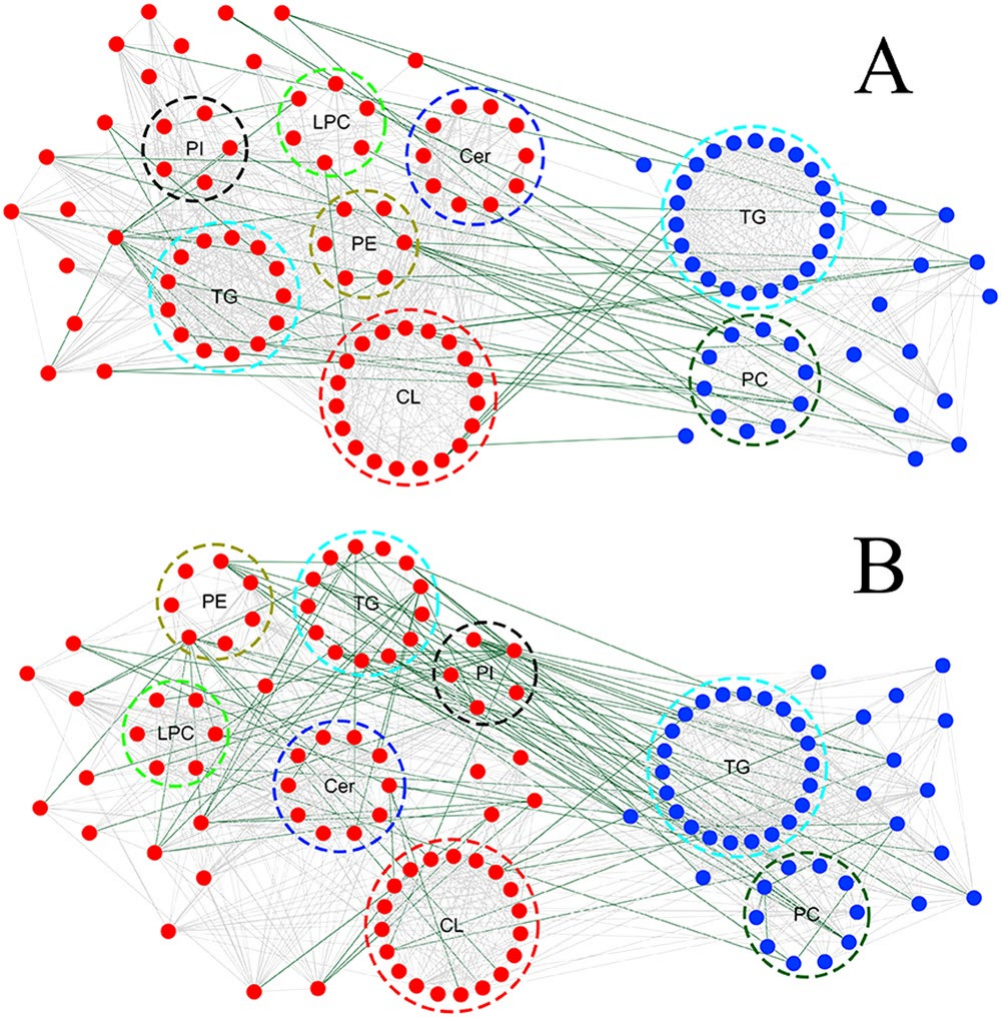
A correlation network of commonly altered lipid classes both within and across LVTA myocardial and serum specimens. Highly correlated lipid species were connected with lines (lipid species were included if they had correlation coefficients >0.6). The dark green and light grey lines represent positive and negative correlations, respectively. The red and blue dots represent lipids in myocardia and sera, respectively. (**A**) ACO-LVTA model; (**B**) CAL-LVTA model; CL: cardiolipin, Cer: ceramide, PE: phosphatidylethanolamine, PI: phosphatidylinositol, TG: triacylglycerol, LPC: lyso-phosphatidylcholine, TG: triacylglycerol, PC: phosphatidylcholine.

First, associations were assessed between commonly altered myocardial and serum lipids. Two correlation networks, representing each of the LVTA models, were constructed according to the correlation coefficients of commonly altered lipids. Figure 4 shows close interactions that were found between commonly altered lipids both within and between myocardial and serum specimens in the two LVTA models. Second, the total relative amounts of differential lipid classes were calculated in the myocardial specimens and compared to the serum specimens. Lysophosphatidylcholine (LPC), phosphatidylethanolamine (PE), phosphatidylinositol (PI) and triacylglycerol (TG) simultaneously disrupted in both myocardial and serum specimens, though with different molecular species and opposite change trends.

### Commonly altered lipid species across two LVTA models

**Figure 5:**
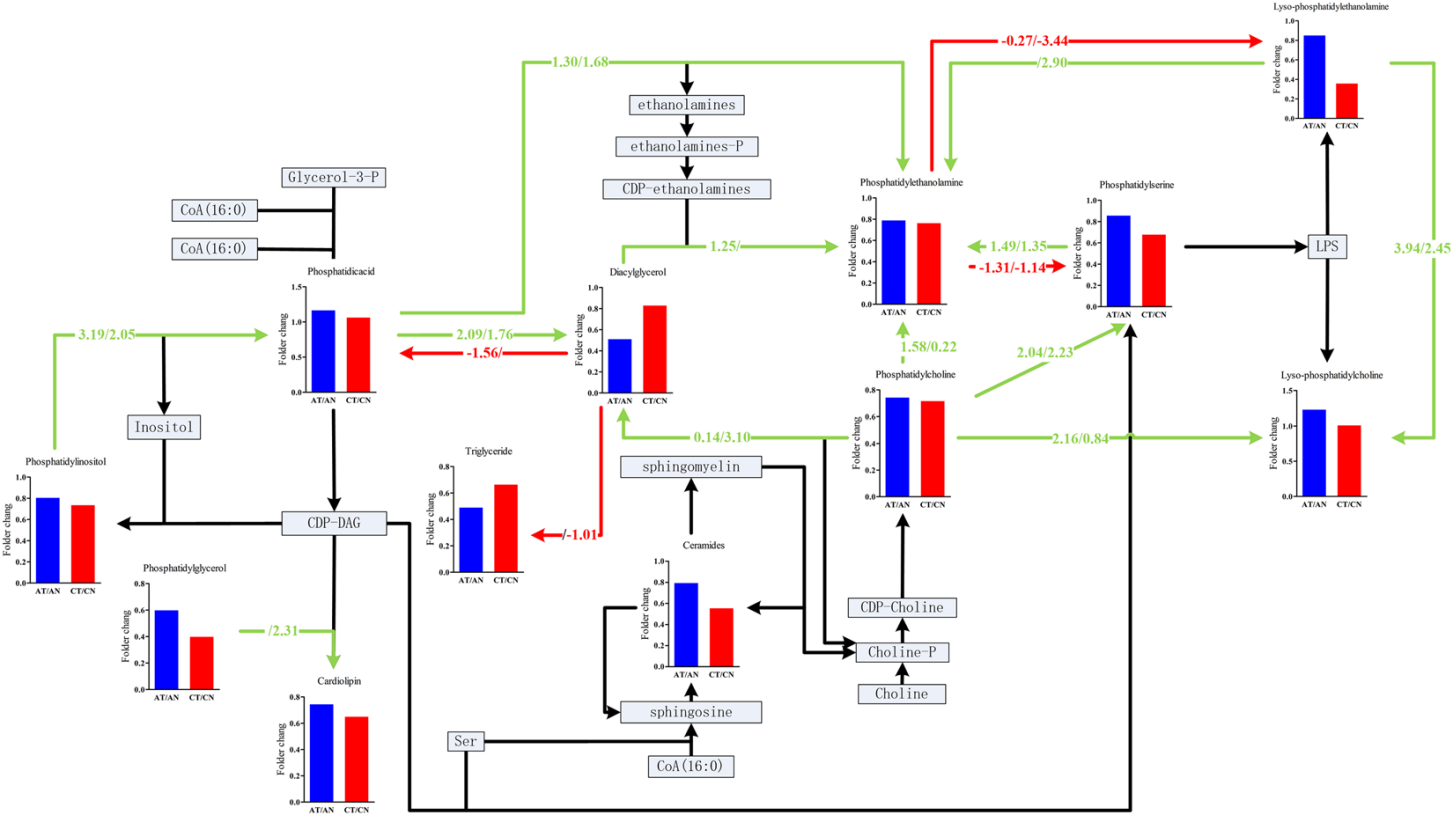
Lipid pathways that were involved in the LVTA models. Fold changes were determined by comparing ratios of the sums of the differential lipid species within a lipid class between LVTA and controls myocardia specimens. The values on each axis denoted *Z*-scores, calculated by Z = *CDF* ^−1^ (1 − *P*), where *CDF* were the cumulative distribution function. *P*-values were calculated using student’s *t*-test between LVTA models and controls and determined the weight of each reaction (each axis). Green lines indicated that the amount of product was greater than the reactant; red lines had the opposite meaning. *Z*-values above slashes were from Model I, while those under slashes were from Model II; *Z*-values greater than 1.645 suggested that a pathway was active.

The 77 lipids with differential abundances identified from the LVTA myocardial specimens belonged to 11 lipid classes. Classes that were down-regulated included: cardiolipin (CL), ceramide (Cer), diacylglycerol (DG), phosphatidylglycerol (PG), lysophosphatidylethanolamine (LPE), phosphatidylserine (PS), PE, PI and TG. Classes that were up-regulated included: LPC and phosphatidic acid (PA). To preliminarily explore LVTA pathophysiological mechanisms that might relate to lipid content, an LVTA lipid metabolic pathway was constructed (Fig. 5). This pathway was built using the differential lipid classes identified above, as well as basic biochemical knowledge. In addition, a previously described bioinformatics method was used to identify active lipid pathways that were altered in the two LVTA models^13^. The *Z*-score of each biochemical pathway was calculated (Supplementary Table 3) and considered active when it was greater than 1.645. Figure 5 shows that several pathways were altered in both LVTA models when compared to controls, including: PC → PS, PI → PA, DG → PA, and LPE → LPC.

### Potential LVTA biomarkers shared by the two models

**Figure 6:**
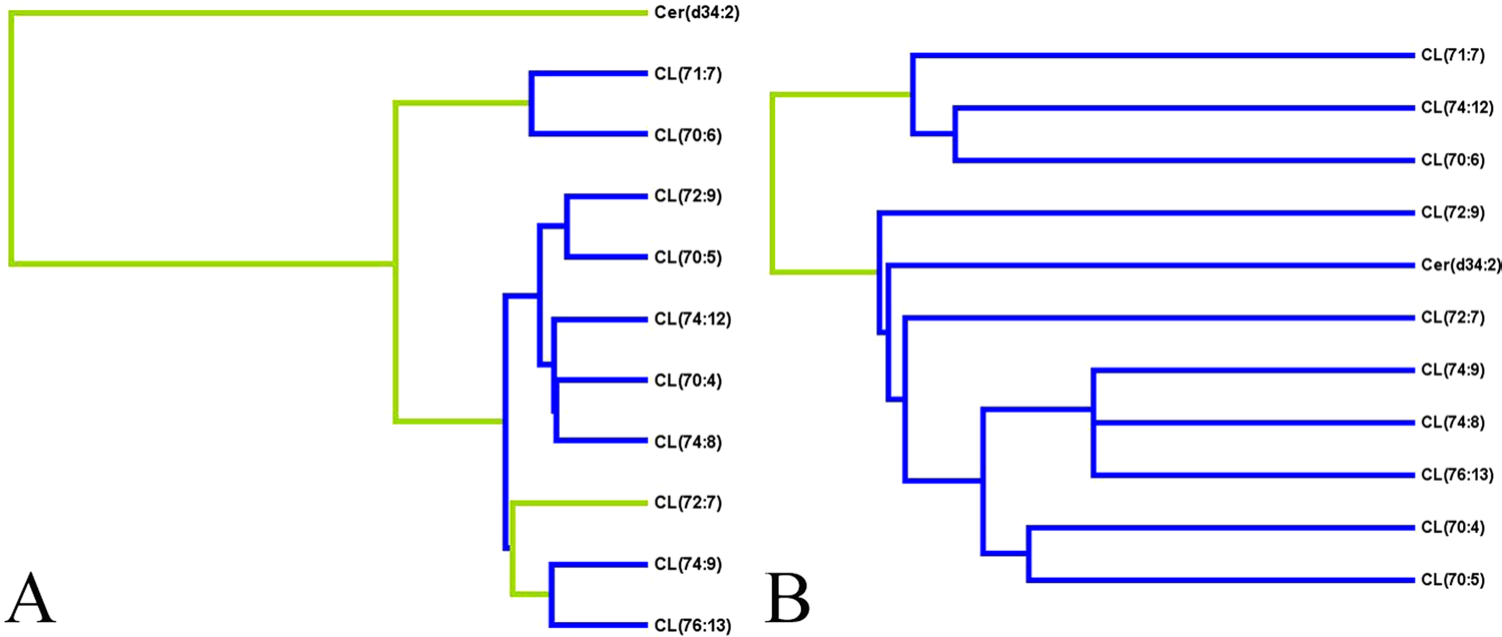
Potential biomarkers common to two LVTA models and their diagnostic potentials. (**A**,**B**) This figure gives HCA graph of 11 lipid species that were altered in both models, (A and B represent Models I and II, respectively).

Differentially abundant lipids may be considered biomarkers of disease when they have high diagnostic potentials, as well as prior associations with pathophysiological mechanisms of disease. Therefore, a two-step process was used to screen potential biomarkers of LVTA common to both models. First, the diagnostic potentials of the commonly altered lipids in the myocardium were analyzed. Eleven lipids that had VIP values greater than 1.5, *P*-values less than 0.01 and area under the curve (AUC) values greater than 0.85 in both LVTA models were selected. These lipid species included: CLs (70:4, 70:5, 70:6, 71:7, 72:7, 72:9, 74:8, 74:9, 74:12, 76:13) and Cer d34:2 (Table 2). After hierarchical cluster analyses by multifactor dimensionality reduction, three lipids were selected, CL 70:5, CL 74:9 and Cer d34:2, representing each branch and a relatively high level of cluster (Fig. 6A and B). To validate the diagnostic values of these three lipids, 5-fold cross-validations of ROC analyses for them in both models were performed. Table 3 shows higher diagnostic accuracy of these three lipids can be achieved in both LVTA models. In addition to having high diagnostic potential, relative contents of these lipids negatively correlated with electrophysiological parameters (Table 2), and occurrences of SCD [for occurrences of SCD: Cer d34:2, relative risk (RR) 0.52, 95% CI 0.29–0.95; CL 70:5, RR 0.58, 95% CI 0.36–0.94; CL 74:9, RR 0.84, 95% CI 0.71–0.99], suggesting that changes in contents of these lipids were crucial to LVTA pathophysiological mechanisms. These data also implied that lower myocardial contents of these lipid species associated with more severe arrhythmias and increased susceptibilities to LVTA-SCD events.

**Table 2:** Correlations between differential lipids and electrophysiological parameters.

Lipid class	Lipid	ACO					CAL				
		AUC	VT	VF	VT + VF	Score	AUC	VT	VF	VT + VF	Score
Ceramides	Cer(d34:2)	0.89			−0.55	−0.54	0.94	−0.46	−0.52	−0.64	−0.70
	Cer(d36:2)	0.85		−0.51	−0.59	−0.49	0.91	−0.52	−0.55	−0.70	−0.70
	Cer(d38:2)	0.79		−0.63	−0.60	−0.50	0.93	−0.54	−0.57	−0.72	−0.78
	Cer(d40:2)	0.76		−0.56	−0.63	−0.62	0.91	−0.52	−0.57	−0.71	−0.73
	Cer(d42:2)	0.79					0.86	−0.49	−0.42	−0.58	−0.59
Cardiolipin	CL(68:2)	0.77	−0.46		−0.55	−0.70	0.97		−0.63	−0.68	−0.63
	CL(68:3)	0.86		−0.48	−0.68	−0.74	0.95		−0.58	−0.63	−0.66
	CL(68:4)	0.76		−0.57	−0.57	−0.50	0.88		−0.49	−0.51	−0.50
	CL(70:4)	0.93		−0.58	−0.73	−0.78	0.98		−0.63	−0.69	−0.67
	CL(70:5)	0.94		−0.60	−0.70	−0.71	0.97		−0.58	−0.64	−0.69
	CL(70:6)	0.88		−0.54			0.92		−0.53	−0.56	−0.56
	CL(70:8)	0.82		−0.63	−0.77	−0.82	0.88	−0.47	−0.41	−0.56	−0.43
	CL(71:7)	0.92		−0.74	−0.61	−0.49	0.92		−0.61	−0.63	−0.56
	CL(71:8)	0.88		−0.70	−0.66	−0.66	0.87		−0.45	−0.55	−0.52
	CL(72:7)	0.86		−0.63	−0.58	−0.54	0.94		−0.62	−0.66	−0.64
	CL(72:8)	0.85		−0.69	−0.72	−0.71	0.91	−0.45	−0.55	−0.66	−0.61
	CL(72:9)	0.96		−0.60	−0.58	−0.57	0.91		−0.58	−0.58	−0.60
	CL(73:7)	0.87		−0.56			0.79				−0.44
	CL(74:12)	0.93		−0.72	−0.74	−0.76	0.92		−0.57	−0.63	−0.59
	CL(74:8)	0.89		−0.57	−0.72	−0.77	0.99	−0.46	−0.69	−0.77	−0.78
	CL(74:9)	0.92		−0.52	−0.58	−0.65	0.98		−0.66	−0.69	−0.71
	CL(76:13)	0.89		−0.50	−0.48	−0.57	0.99	−0.44	−0.63	−0.70	−0.69
Lyso-phosphatidylcholine	LPC(24:0)	0.77					0.83		0.53	0.59	0.67
Phosphatidylglycerol	PG(36:4)	0.83			−0.60	−0.58	0.89	−0.43	−0.58	−0.66	−0.63
Phosphatidylinositol	PI(38:6)	0.81			−0.46	−0.64	0.79	−0.44		−0.44	
Phosphatidylserine	PS(42:3)	0.77					0.90	−0.44	−0.62	−0.70	−0.70

Notes: AUC: area under curve. VT: ventricular tachycardia duration. VF: ventricular fibrillation duration. VT + VF: the total duration of ventricular tachycardia and ventricular fibrillation. Score: the arrhythmia scores were calculated based on the Lambeth Conventions principles; in all columns except AUC, the data in the table were the correlation coefficients between the relative amounts of lipids with these parameters.

**Table 3:** 5-fold cross-validations of ROC analyses of three potential lipid biomarkers.

	ACO-T		CAL-T	
	AUC	Variance	AUC	Variance
CL(70:5)	0.944	0.002	0.983	0.001
CL(74:9)	0.894	0.002	0.966	0.001
Cer(d34:2)	0.900	0.002	0.906	0.002

Notes, AUC: area under curve, ACO-T, CAL-T, the AUC values to diagnose ACO-T and CAL rats from their respective controls.

## Discussion

LVTA is the most frequent electrophysiological etiology leading to cases of SCD. It is of interest to identify lipid profiles of LVTA-SCDs and to determine whether they are shared when caused by different pathological conditions. MI and myocardial ion channel diseases are two main causal disorders for SCD^14^. Our team has previously developed two rat models that faithfully represented human disorders^7^. The first model was produced by aconitine, the result of which mimicked cardiac ion channel diseases and subsequent LVTA-SCD. The second was a CAL-stimulated myocardial infarction model, which mimicked coronary heart disease-related LVTA-SCD; the most common form of structural heart disease-related SCD. Together, these two models represented common LVTA pathologies and were useful for studying lipid metabolism of this disease. It is important to note that each control population received the same diets, housing conditions and treatments leading to the same pathological conditions as their test counterparts. The difference between the controls and the test animals was simply that the controls did not experience LVTA. Thus, LVTA was an independent factor contributing to the observed lipid differentials between SCD groups and their respective controls.

Additionally, the echocardiographic features of both LVTA models were similar. Subjects from both groups experienced ventricular premature beats, followed by VT and VF with subsequent LVTA-induced mortality; ECG parameters between the groups were not significantly different (*P* > 0.05, Table 1). Hemodynamic analyses showed similar decreases of left ventricular function in the LVTA models (Fig. 2).

### Interpretation of common differential lipid profiles

The PLS-DA plots of lipidomes showed clear separations between the LVTA models versus controls, across both myocardial and serum specimens (Supplementary Fig. 1s). This indicated a profound disruption of the lipidome in the LVTA models. Theoretically, these alterations were caused by LVTA because it was the only variable in both models after adjusting for their respective controls.

From the two LVTA models, 77 and 46 common lipids of differential abundances were identified in the myocardia and sera, respectively. In the myocardia, the commonly down-regulated lipid classes included: CL, Cer, DG, PE, PG, PI, TG, LPE and PS. Commonly up-regulated classes included: LPC and PA. Biochemical pathways which were active in LVTA myocardia, included: PC → PS, PI → PA, DG → PA, and LPE → LPC. Alterations of these pathways suggest that relevant enzymes activities would also be altered, as shown in previous work^13^. These enzymes might serve as therapeutic targets for LVTA-SCD.

Most of the down-regulated lipid classes were phospholipids. PE and PS are two major components of cell membranes^9, 15^. Declines or changes in species compositions of these phospholipids would affect cell membrane curvatures and stabilities. Changes in composition would dramatically alter electrophysiological stabilities of cell membranes by influencing membrane potentials and ion transport functions, ultimately inducing lethal arrhythmias. Additionally, LPC has been shown to accumulate in ischemic myocardia and induce arrhythmia-related electrophysiological alterations^9, 16, 17^. Furthermore, both PE and PS have influenced apoptotic and necrotic mechanisms of myocardial cells after myocardial ischemic and inflammatory events^18^. Thus, LVTA-induced down-regulations of these two phospholipids may influence myocardial cell death regulatory mechanisms.

Notably, 19 different molecular species in the CL lipid class were simultaneously down-regulated in LVTA myocardial specimens, indicative of key roles for this class in LVTA pathophysiologies. CLs are also known as ‘heart phospholipids’ and are responsible for maintaining the functions and morphologies of mitochondria^19^. Cardiomyocytes are mitochondria-rich; mitochondrial dysfunction has closely correlated with lethal arrhythmias, especially ventricular fibrillation^3, 20, 21^. Decreased quantities of CL species in LVTA myocardia may lead to mitochondria dysfunction^11^, and subsequent lethal arrhythmias. In addition, CL levels were reduced in both failure and diabetic myocardia, where they regulated mitochondrial apoptotic mechanisms^21, 22^. Therefore, decreased CL levels in the models presented here may regulate LVTA-induced myocardial necrosis. PG species are precursors to CL species. LVTA-induced declines in PG levels may partially explain concomitant CL declines; together these reductions may exacerbate the outcomes of lowered CL levels during LVTA events.

Cer levels have been associated with cardiovascular events, and have long been considered drivers of apoptosis^23, 24^. In contrast, data presented here showed that Cer levels were reduced in myocardia that experienced LVTA. One explanation for this could be that Cer is derived from dihydroceramide, which is inhibited by oxidative stress and hypoxia. Oxidative stress and hypoxia are MI complications that are also present in trembling myocardia^25^. Thus, decreased myocardial Cer abundances in LVTA subjects suggested that these cardiomyocytes experienced even more severe hypoxias and oxidative stress events than controls. This phenomenon could partially explain how LVTA models become more vulnerable to LVTA-related SCD than controls.

TG species are considered the major energy source for a working myocardium. TG-related fatty acids account for approximately 40% of myocardial oxygen consumptions^26^. Deregulated TG metabolisms and altered cardiac TG content are associated with impaired heart functions^27^. In a trembling heart, fat mobilization was enhanced due to sympathetic excitation, which used more TG to satisfy increased energy requirements. Our previous study found that five free fatty acids were simultaneously down-regulated in LVTA myocardia^7^. Therefore, decreased quantities of TG and fatty acids suggested that energy requirements and consumption were increased during LVTA, and that resultant lowered energy levels contributed to subsequent collapses of trembling hearts.

Surprisingly, PC, PE, PI and TG lipid classes, which were down-regulated in the myocardia, were up-regulated in the corresponding sera. Given that these phospholipids are critical membrane components, they may be released into the blood by cardiomyocytes during LVTA-induced cell membrane disintegration events. Furthermore, increased fat mobilization has led to increased release of free fatty acids into the blood during LVTA, driving the liver to synthesize more TG^28^. This could result in relatively high levels of these species in LVTA-exposed serum specimens.

### Common potential biomarkers

CL 70:5, CL 74:9 and Cer d34:2 were identified as a potential suite of biomarkers that could indicate myocardial LVTA in these models. The fatty acid branched chains were 18:2/16:0/18:1/18:1 in CL 70:5, 18:2/18:2/20:3/18:2 in CL 74:9 and d16:1/18:1 in Cer d34:2, respectively (Supplementary Table 1 and Fig. 2). The majority of species were (18:2) and (18:1), which were consistent with previous data showing that major species of CL fatty acid chains included linoleic (18:2) and oleic (18:1) acids^29^. The remaining three fatty acid chains were palmitic acid (16:0), eicosatrienoic acid (20:3) and palmitoleic acid (16:1). Together, the lipid species were significantly altered, with high diagnostic values to identify LVTA events induced by either MI or myocardial ion channel diseases. Moreover, the species closely correlated with electrophysiological parameters of the subjects and associated with occurrences of SCD. Decreased abundances of these species could accurately reflect the severity of arrhythmias and indicate a high probability of future SCD events. As discussed above, downregulations of both of CL and Cer species in the myocardia were critical to LVTA pathophysiological mechanisms.

### Limitations

There were several limitations of this work. First, this is a descriptive study. Future studies must confirm the metabolic mechanism that could potentially be altered by LVTA, identified here. Additionally, the pathological disorders modeling LVTA in this study occurred either acutely or in the early stage of the disease course. Thus, metabolic LVTA feature that result from chronic conditions such as diabetes, heart failure and chronic myocardial ischemia should be considered in future studies.

## Conclusions

In the present study, a shared lipid profile was identified in myocardia and sera that were exposed to MI- and myocardial ion channel disease-induced LVTA events. CL 70:5, CL 74:9, and Cer d34:2 were identified as potential lipid biomarkers of LVTA in the myocardia of both models. These three lipid species had high diagnostic potentials in the models, and closely correlated with electrophysiological parameters of the subjects. These results provided new insights into the pathophysiological processes of LVTA and may offer therapeutic targets for LVTA-SCD induced by independent mechanisms.

## Materials and Methods

### Animals

This study was approved by the Medical Animal Care & Welfare Committee at Shantou University Medical College. All procedures were carried out in accordance with the Guide for Care and Use of Laboratory Animals of Shantou University Medicine College.

Adult male Sprague-Dawley rats (weight range, 250–400 g) were used in this study. Animals were supplied by the Animal Research Center of Shantou University Medical College. All rats were kept in plastic cages at room temperature with 12-h light/dark cycles and a relative humidity of 50–60%.

### Establishment of rat LVTA-SCD models

Two rat LVTA-SCD models were established as previously described^7^ and are briefly described as follows:

Model I: rats were anesthetized by pentobarbital sodium and lead II ECG rates were monitored using a Biological Functional Experimental System (Chengdu Taimeng Co. Ltd, China). Subsequently, rats were tail-vein injected with aconitine (100 μg/mL dissolved in saline, 100 μL for each rat). The rats that developed typical VT and VF, and then subsequently died, were defined as the ACO-LVTA group. Other rats, injected with relatively lower dosages of aconitine (10 μg/mL in saline, 50 μL for each rat), died of lethal bradycardiac arrhythmias and served as controls (the ACO-N group).

Model II: rats were anesthetized and monitored by ECG measurements as in Model I. CAL was performed to induce acute MI. The rats that developed VT, subsequent VF, and died soon thereafter, were designated as the CAL-LVTA group. Those who did not develop VT or VF, but instead developed bradycardia and died of severe atrioventricular block, were controls (the CAL-N group). To assess the severity of arrhythmias, ECG parameters were recorded and arrhythmia scores were calculated as previously described^30^.

To monitor changes in left ventricular functions during LVTA events, cardiac hemodynamics were measured. Briefly, after the rats were anesthetized and monitored by ECG, fluid-filled polyethylene catheters, which connected to the above Experimental System, were catheterized into right common carotid arteries and then threaded into left ventricular cavities. The catheters were used to record LVSP and maximum rate of ventricular pressure increase (+dP/dt_max_) measurements.

Immediately after subject deaths, blood samples were drawn from caval veins. Serum specimens were separated with centrifugation at 3000 rpm for 10 min and stored at −80 °C. Whole hearts were harvested on ice and then stored at −80 °C.

### Myocardial pre-treatments prior to lipidomics

Myocardial specimens were processed as previously described^31^. For each experimental subjects, ten milligrams of the left ventricular myocardium, located two millimeters from the apex were excised, mixed with 500 μL of purified water, and then homogenized by a multi-sample organ grinder (Tissuelyser-48, Shanghai Jingxin Technology Company, China) at 60 Hz for 90 s. Homogenates were transferred into glass centrifuge tube and 1.5 mL aliquots of dichloromethane/methanol (2/1, v/v) were added along with two internal standards, LPC (12:0) and PC (11:0/11:0). Specimens were vortexed for one minute and resulting homogenates were centrifuged at 3000 rpm for 15 min. The bottom layer was transferred into another glass tube and 1.5 mL aliquots of dichloromethane/ methanol (2/1, v/v) were added. The upper liquid layers was processed once again, as above. The two bottom liquid layers were combined and freeze-dried in a freeze concentration centrifugal dryer (LNG-T98, Taicang City Huamei Biochemistry Instrument company, China). The resultant powder was dissolved in isopropanol/methanol (1/1, v/v) and stored at −20 °C for lipidomic analyses.

### Serum pretreatments prior to lipidomics

One hundred microliters of serum specimens were mixed with 100 μL of purified water in glass tubes. Next, 1.5 mL aliquots of dichloromethane/methanol (2/1, v/v) were added and vortexed for one minute. The homogenates were then treated with the same processes as described above for the myocardial specimens.

### Lipid profiling analyses

Lipidomic analyses were conducted on an Ultimate-3000 UPLC system coupled to a Q Exactive hybrid quadrupole-Orbitrap MS system (Thermo Scientific). A 100 × 2.1 mm hypersil GOLD 1.9-μm C18 column (Thermo Scientific) was used. The column temperature was maintained at 45 °C. Eluent A (60% acetonitrile and 40% water containing 10 mmol/L ammonium formate) and eluent B (10% acetonitrile and 90% isopropanol containing 10 mmol/L ammonium formate) were used. The flow rate was 0.35 mL/min and the injection volume was four microliters. The gradient was 40–100% eluent B over 14.5 min, 100% eluent B over 14.5–16.5 min, 100–40% eluent B over 16.50–16.51 min, 40% eluent B over 16.51–20 min. All samples were kept at 15 °C during analyses. Detailed descriptions of the MS detection processes are described in the supplementary information.

### Data processing and statistical analyses

First, all raw data were acquired using the software Xcalibur (version 3.0, Thermo Scientific). LipidSearch (Version 4.0, Thermo Scientific) was used for lipid identification and quantification. Lipids were identified according to their exact masses, retention times, and the patterns of their precursor ions and MS2. For each subject, acquired data were used to construct a raw data matrix, which consisted of sample information as well as lipid species and their classifications, retention times, charge-mass ratios and peak areas.

Second, data matrices were imported into SIMCA software (version 13.0, Umetrics) to establish the PLS-DA model with UV scaling, for multivariate analyses. To evaluate the quality of the PLS-DA model, 7-fold cross validation and 200 times response per mutation assays were performed. Parameters of the models, including: R^2^X, R^2^Y, and Q^2^Y, and the R^2^Y-, Q^2^Y-intercepts, were analyzed to ensure the quality of the multivariate models and to avoid the risk of over-fitting.

Differentially abundant lipids were screened according to VIP values in the PLS-DA model. Additionally, *P*-values were measured with student’s *t*-tests between the LVTA models and their respective controls. *P*-values less than 0.05, or VIP values greater than one were considered to be significantly different^32^.

The overall differences of the common differential lipids between the two LVTA groups were analyzed by the MeV software package (version 4.5.1). The data were presented as a heatmap.

Correlation coefficients among the commonly altered lipids were calculated using SPSS 17.0 (SPSS Inc., Chicago, USA) and then two interactive networks were constructed using Cytoscape software (version 3.4.0) to analyze the interactions of these lipids, and to partially validate the myocardial lipidomes in corresponding serum samples.

Diagnostic potentials of altered lipid species were analyzed by a receiver operating characteristic curves generated with MedCalc software. The optimal cut-off point, the AUC, and the sensitivity and specificity values were determined for each lipid. Hierarchical cluster analyses were performed with multifactor dimensionality reductions^33^. Associations of potential biomarkers with SCD were analyzed with binary logistic regression methodologies, in which RR and 95% confidence intervals were calculated.

## Electronic supplementary material

Supplemental information
