# Supplementary material for: Common lipid features of lethal ventricular tarchyarrhythmias (LVTAs) induced by myocardial infarction and myocardial ion channel diseases
Source: Sci Rep. 2017 Jun 26;7:4220. doi: 10.1038/s41598-017-04620-w (PMC5484696; doi:10.1038/s41598-017-04620-w)
Supplement: Supplementary file 1 — Supplemental information [file 41598_2017_4620_MOESM1_ESM.pdf]

Supplemental Information

A common lipid feature of lethal ventricular tarchyarrhythmias (LVTAs) induced by myocardial infarction and myocardial ion channel disease

Jiayan Wu[1]. Qian Wu[2]. Dian Wang[1*]. Jing Kong[1]. Xingxing Wang[3]. Xiaojun Yu[1].

[1] Department of Forensic Medicine, [3] 2nd Affiliated Hospital, Shantou University Medical College, Shantou, 515041, China.

[2] Shanghai Center for Bioinformation Technology, Shanghai, 201203, China.

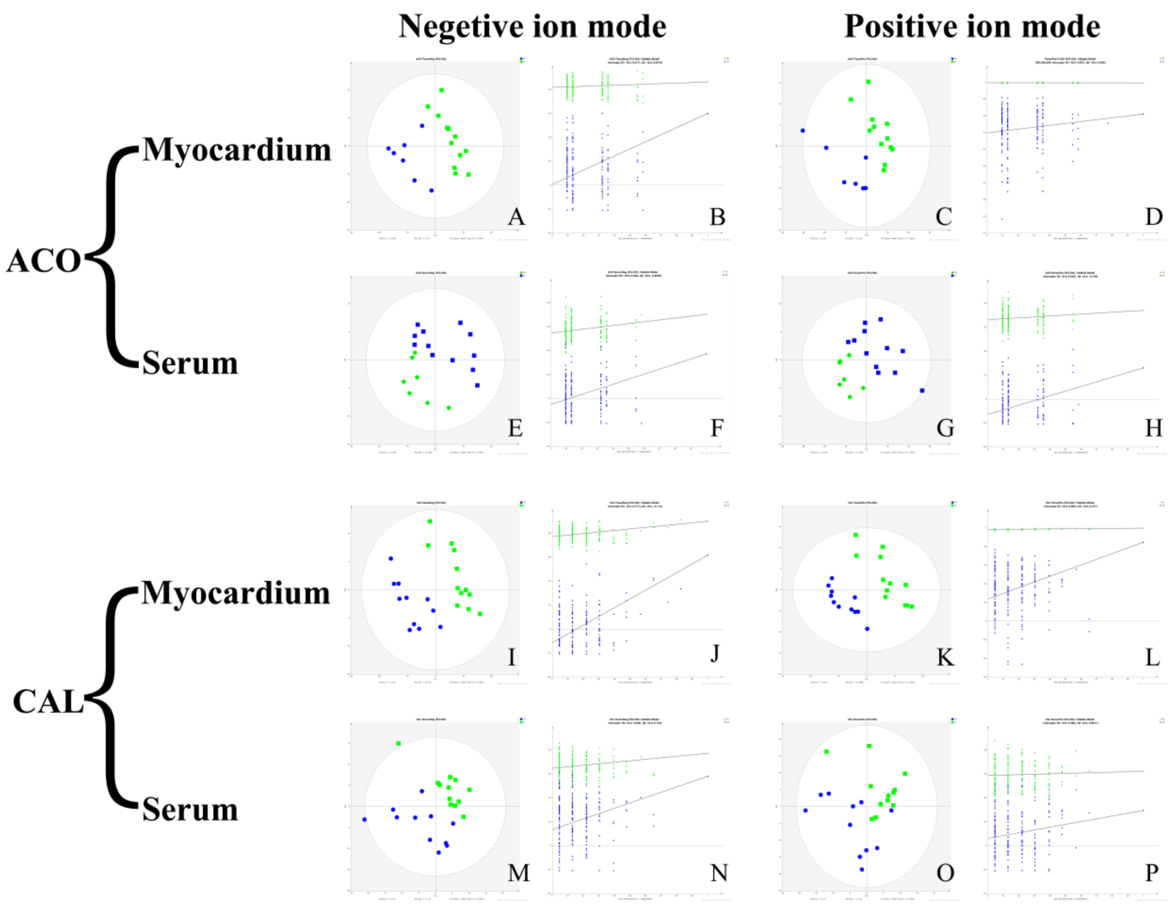

Supplementary Figure 1. The PLS-DA score plots and validation graphs of lipid profile of two rat LVTA models. The lipid profiles in the myocardia and serum were analyzed by both negative and positive ion modes. Apparent separations between the LVTA groups and their respective controls were observed both in the myocardia and serum on the left figures of each ion mode. The right figures of each ion mode showed almost no over-fitting in each groups. ACO (Model I): aconitine injection. CAL: Model II (coronary artery ligation).

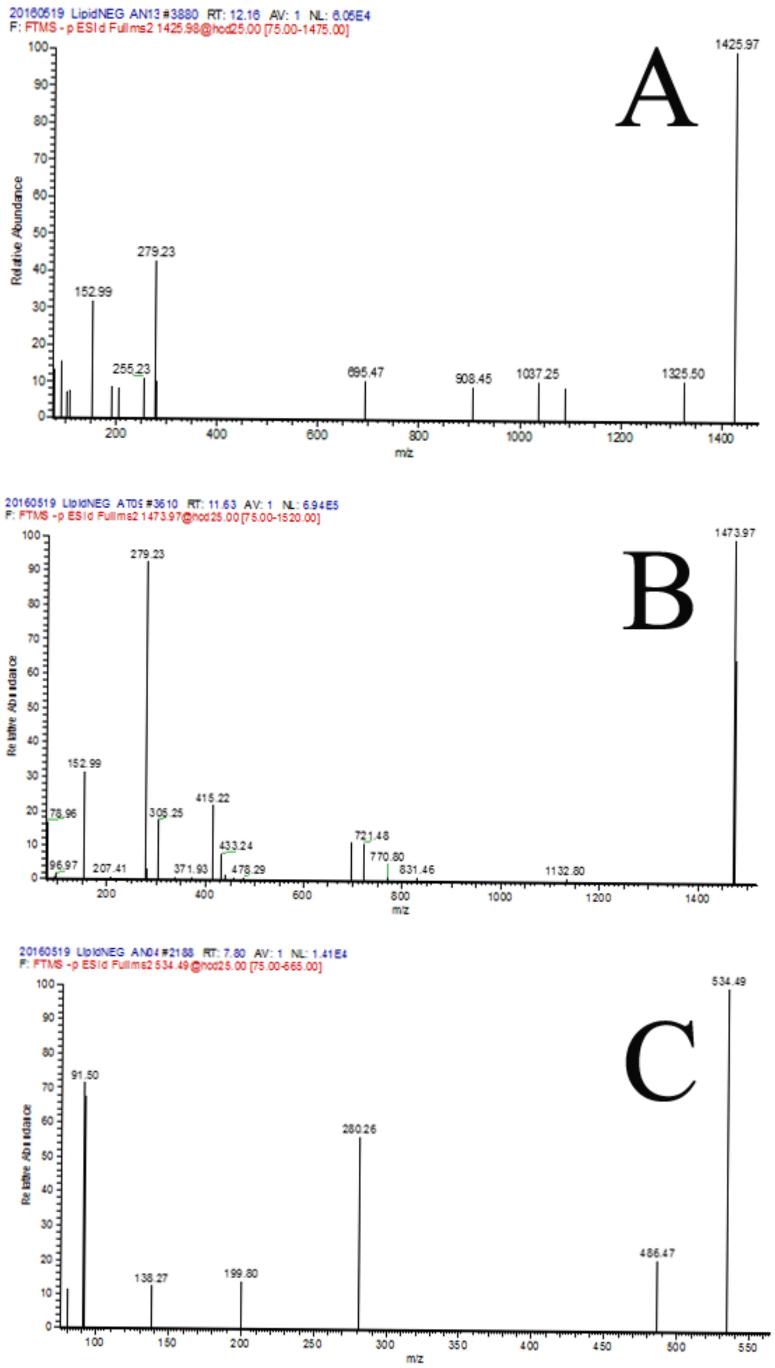

Supplementary Figure 2. MS/MS fragment spectra of three potential lipid biomarkers. A, CL (18:2/18:2/16:0/18:1) or CL (70:5); **B,** CL (18:2/18:2/20:3/18:2) or CL (74:9); **C,** Cer (d16:1/18:1) or Cer (d34:2).

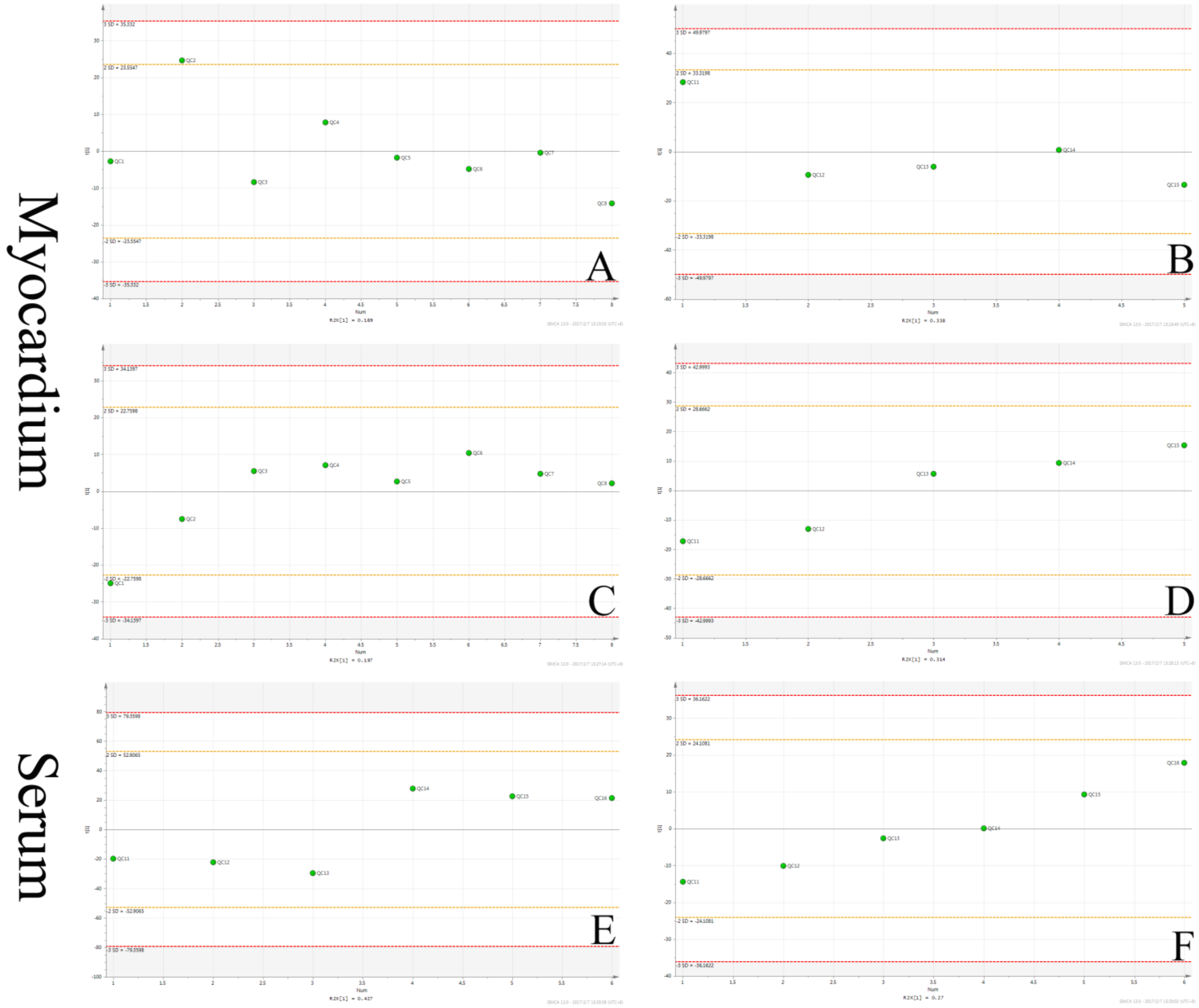

Supplementary Figure 3. One dimensional PCA-X of QC: A, B, data from the positive mode of the myocardia; C, D, data from the negative mode of the myocardia; E, F, data from the positive and negative modes of the serum, respectively. QC 1- 8 samples were run in succession before the experimental sample detection; QC 11-15 samples (11-16 samples in serum) were run after every 10 injection (experimental samples).

Supplementary Table 1. Seventy-seven common significant lipids with the same change tendency in the two LVTA groups

Lipid classes	Lipids	Ion modes	Fatty Acids	Rt (min)	Model I (ACO)			Model II (CAL)		
					VIP	*P*-value	FC	VIP	*P*-value	FC
Ceramides	Cer(d34:1)	neg	(d18:1/16:0)	8.54	1.19	7.22E-02	0.85	1.72	8.10E-05	0.56
	Cer(d34:2)	neg	(d16:1/18:1)	7.82	1.70	2.73E-03	0.62	1.61	6.38E-04	0.48
	Cer(d36:1)	neg	(d18:1/18:0)	9.26	1.18	6.16E-02	0.82	1.78	2.54E-05	0.61
	Cer(d36:2)	neg	(d16:1/20:1)	8.63	1.70	1.22E-02	0.64	1.59	3.38E-04	0.59
	Cer(d38:1)	pos	(d18:1/20:0)	9.89	1.14	8.72E-02	0.86	1.84	3.62E-05	0.60
	Cer(d38:2)	neg	(d16:1/22:1)	9.33	1.38	4.86E-02	0.76	1.74	1.33E-04	0.44
	Cer(d40:2)	pos	(d18:2/22:0)	9.93	1.26	2.88E-02	0.81	1.79	8.50E-05	0.51
	Cer(d42:2)	pos	(d18:2/24:0)	10.54	1.30	1.18E-02	0.73	1.61	6.54E-04	0.59
	Cer(d42:3)	pos	(d18:2/24:1)	9.93	1.27	5.20E-02	0.85	1.82	1.02E-04	0.54
	Cer(d42:3)	neg	(d18:1/24:2)	9.94	1.20	5.85E-02	0.82	1.77	9.42E-05	0.53
Cardiolipin	CL(68:2)	neg	(18:1/16:0/16:0/18:1)	12.60	1.19	3.70E-02	0.79	1.98	1.71E-05	0.54
	CL(68:3)	neg	(18:2/16:0/18:1/16:0)	12.34	1.63	1.13E-02	0.71	1.86	1.97E-04	0.53
	CL(68:4)	neg	(18:2/16:0/16:0/18:2)	12.10	1.56	4.63E-02	0.77	1.59	1.98E-03	0.58
	CL(68:6)	neg	(18:2/18:2/18:2/14:0)	11.51	1.37	1.04E-01	0.82	1.34	1.64E-02	0.75
	CL(70:4)	neg	(18:2/16:0/18:1/18:1)	12.37	2.00	7.13E-04	0.67	2.00	1.78E-05	0.55
	CL(70:5)	neg	(18:2/18:2/16:0/18:1)	12.16	2.05	7.04E-04	0.69	1.86	1.12E-04	0.56
	CL(70:6)	neg	(18:2/16:0/18:2/18:2)	11.84	1.75	5.44E-03	0.75	1.61	4.04E-04	0.64
	CL(70:8)	neg	(16:2/18:2/18:2/18:2)	11.26	1.52	2.89E-02	0.79	1.63	7.60E-04	0.71
	CL(71:7)	neg	(17:1/18:2/18:2/18:2)	11.67	1.93	1.90E-03	0.76	1.71	8.39E-05	0.69
	CL(71:8)	neg	(15:0/18:2/20:4/18:2)	11.41	1.72	1.07E-02	0.74	1.45	1.01E-03	0.70
	CL(72:7)	neg	(18:2/18:1/18:2/18:2)	12.24	1.60	6.92E-03	0.82	1.87	1.71E-05	0.65
	CL(72:8)	neg	(18:2/18:2/18:2/18:2)	11.59	1.61	3.11E-02	0.83	1.70	9.75E-05	0.76
	CL(72:9)	neg	(18:3/18:2/18:2/18:2)	11.32	2.00	3.77E-04	0.68	1.65	1.82E-04	0.65
	CL(73:7)	neg	(19:1/18:2/18:2/18:2)	12.06	1.79	9.10E-03	0.62	1.06	2.45E-02	0.63
	CL(74:12)	neg	(18:3/16:1/18:2/22:6)	11.28	2.00	1.57E-03	0.75	1.79	1.42E-04	0.62

	CL(74:8)	neg	(20:2/18:2/18:2/18:2)	11.87	1.85	6.77E-03	0.69	2.14	1.06E-06	0.64
	CL(74:9)	neg	(18:2/18:2/20:3/18:2)	11.63	1.86	3.55E-03	0.73	2.04	5.22E-06	0.67
	CL(76:12)	neg	(18:2/18:2/18:2/22:6)	11.35	1.24	6.51E-02	0.75	1.40	1.24E-02	0.75
	CL(76:13)	neg	(18:3/20:4/18:2/20:4)	11.44	1.69	8.05E-03	0.78	2.07	6.30E-06	0.70
Diacylglycerol	DG(34:2)	pos	(16:0/18:2)	9.13	1.31	3.84E-02	0.49	1.52	7.94E-01	0.87
	DG(36:2)	pos	(18:0/18:2)	9.78	1.10	1.52E-01	0.54	2.03	7.64E-01	0.86
	DG(36:3)	pos	(16:0/20:3)	9.25	1.17	1.22E-01	0.50	1.32	7.03E-01	0.71
	DG(38:3)	pos	(20:1/18:2)	9.74	1.26	7.45E-02	0.50	1.04	8.02E-01	0.88
Fatty acid	FA(22:5)	neg	(22:5)	2.84	1.03	1.53E-01	0.79	0.90	4.76E-02	0.70
Lyso-phosphatidylcholine	LPC(18:2)	pos	(18:2)	1.79	1.96	2.14E-05	2.52	1.23	4.16E-01	1.16
	LPC(19:0)	pos	(19:0)	3.34	1.13	5.28E-02	0.73	1.08	8.18E-02	0.74
	LPC(22:0)	pos	(22:0)	5.27	0.93	1.15E-02	1.25	0.94	1.88E-02	1.30
	LPC(24:0)	pos	(24:0)	6.38	1.58	2.60E-02	1.21	1.80	4.60E-03	1.31
	LPC(37:5)	pos	(37:5)	9.55	1.17	7.99E-02	0.85	1.28	6.19E-03	0.79
	LPC(P-16:0)	pos	(16:0p)	2.46	1.20	5.90E-02	0.76	1.38	4.46E-03	0.49
Lyso-phosphatidylethanolamine	LPE(19:0)	neg	(19:0)	3.49	1.00	1.09E-01	0.82	1.59	8.20E-04	0.32
	LPE(P-18:0)	neg	(18:0p)	3.61	1.18	1.24E-01	0.88	1.58	8.70E-04	0.39
Phosphatidicacid	PA(46:7)	neg	(26:3/20:4)	8.06	1.07	1.18E-01	1.17	1.27	5.09E-01	1.06
Phosphatidylcholine	PC(26:0)	pos	(10:0/16:0)	5.99	1.25	3.10E-01	0.80	1.39	4.82E-02	0.53
	PC(37:1)	neg	(18:1/19:0)	9.66	1.55	2.38E-02	0.74	1.21	1.79E-01	0.90
	PC(40:4)	neg	(18:0/22:4)	9.19	1.36	8.43E-02	0.68	1.30	7.32E-03	0.72
Phosphatidylethanolamine	PE(44:10)	neg	(22:4/22:6)	7.48	1.09	1.28E-01	0.79	1.21	9.53E-03	0.68
	PE(44:5)	pos	(24:1/20:4)	9.94	1.17	2.15E-01	0.83	1.30	5.97E-03	0.79
	PE(O-36:1)	neg	(18:0e/18:1)	9.88	1.11	1.08E-01	0.73	1.23	5.20E-03	0.65
	PE(P-36:0)	neg	(18:0p/18:0)	10.32	1.00	2.56E-01	0.91	1.41	1.37E-03	0.75
	PE(P-36:4)	pos	(16:0p/20:4)	8.37	1.01	7.64E-01	0.98	1.06	1.10E-01	0.83
	PE(P-38:3)	pos	(20:1p/18:2)	9.23	1.65	3.91E-03	0.58	1.29	1.01E-01	0.77
	PE(P-40:5)	pos	(18:0p/22:5)	9.04	1.44	1.76E-01	0.70	1.38	1.72E-01	0.86
Phosphatidylglycerol	PG(36:4)	pos	(16:0/20:4)	6.81	1.64	1.99E-02	0.12	1.76	1.78E-04	0.20
	PG(36:4)	neg	(18:2/18:2)	6.74	1.30	6.13E-02	0.86	1.65	2.28E-04	0.45
	PG(38:5)	neg	(16:0/22:5)	7.21	1.06	1.12E-01	0.82	1.41	2.52E-03	0.54

Phosphatidylinositol	PI(38:5)	neg	(18:1/20:4)	7.14	1.11	1.03E-01	0.87	0.98	2.56E-02	0.81
	PI(38:6)	neg	(16:0/22:6)	6.75	1.26	3.93E-02	0.81	1.13	1.51E-02	0.72
	PI(40:4)	neg	(18:0/22:4)	8.37	1.17	1.73E-01	0.88	1.50	2.84E-03	0.81
	PI(40:8)	neg	(20:4/20:4)	6.21	1.29	5.48E-02	0.69	1.14	1.54E-02	0.59
	PI(61:13)	neg	(37:6/24:7)	10.15	1.24	2.62E-02	0.77	1.14	1.18E-01	0.73
Phosphatidylserine	PS(37:0)	neg	(18:0/19:0)	9.32	1.05	1.33E-01	1.16	1.03	7.57E-01	1.02
	PS(39:4)	neg	(21:2/18:2)	6.77	1.39	1.04E-01	0.62	1.05	4.80E-02	0.52
	PS(42:3)	neg	(18:1/24:2)	10.58	1.45	3.86E-02	0.78	1.79	1.86E-05	0.49
Triglyceride	TG(44:0)	pos	(16:0/12:0/16:0)	12.04	1.16	2.77E-02	0.86	1.07	1.75E-01	0.46
	TG(54:7)	pos	(16:0/18:2/20:5)	11.71	1.19	1.22E-01	0.60	1.22	6.83E-01	0.68
	TG(56:5)	pos	(18:0/16:0/22:5)	12.60	1.22	1.93E-01	0.44	1.18	5.70E-01	0.57
	TG(56:7)	pos	(18:1/18:1/20:5)	12.06	1.24	1.69E-01	0.40	1.22	9.11E-01	0.92
	TG(56:9)	pos	(20:5/18:2/18:2)	11.38	1.25	3.11E-01	0.48	1.08	6.94E-01	0.65
	TG(58:4)	pos	(22:0/18:2/18:2)	13.04	1.22	1.92E-01	0.37	1.29	6.24E-01	0.59
	TG(58:7)	pos	(18:0/18:1/22:6)	12.46	1.20	2.24E-01	0.44	1.12	8.63E-01	0.87
	TG(59:3)	pos	(18:1/18:2/23:0)	13.38	1.32	3.56E-01	0.59	1.12	9.13E-01	0.92
	TG(60:3)	pos	(18:0/18:2/24:1)	13.50	1.21	2.38E-01	0.46	1.11	6.11E-01	0.67
	TG(60:4)	pos	(18:1/18:2/24:1)	13.27	1.21	2.27E-01	0.36	1.03	5.53E-01	0.48
	TG(60:5)	pos	(24:1/18:2/18:2)	13.02	1.22	1.92E-01	0.33	1.10	8.73E-01	0.84
	TG(62:4)	pos	(26:1/18:1/18:2)	13.51	1.24	2.85E-01	0.45	1.11	6.31E-01	0.56
	TG(O-38:2)	pos	(8:0e/12:0/18:2)	10.26	1.16	1.08E-01	0.59	1.02	6.98E-02	0.39

Notes: Fatty Acid: the fatty acid branch chains substituting for the hydroxyl or amino in lipid. Rt: retention time. CAL: Model II (coronary artery ligation). ACO (Model I): aconitine injection. VIP: variable important in projection. *P*-value: significance of a t-test between the LVTA groups and their respective controls. FC: folder change, the ratio of relative amount of an individual differential lipid in the LVTA group to that of its control.

Supplementary Table 2. Forty-six common differential lipids with the same change tendency in two LVTA models in the serum

Lipid Classes	Lipids	Ion	Fatty Acids	Rt (min)	CAL			ACO		
					VIP	*P*-value	FC	VIP	*P*-value	FC
Digalactosyl diacylglycerol	DGDG(35:8)	neg	(13:2/22:6)	2.51	1.76	8.41E-02	0.41	1.47	2.31E-01	0.58
Lyso-phosphatidylcholine	LPC(17:0)	pos	(17:0)	2.58	1.03	5.93E-01	1.14	1.41	1.00E-01	1.40
	LPC(18:0)	pos	(18:0)	2.99	1.13	1.57E-01	0.70	1.15	5.16E-01	0.61
	LPC(20:0)	pos	(20:0)	4.27	1.32	3.86E-01	1.21	1.68	3.04E-02	1.60
	LPC(23:0)	neg	(23:0)	6.14	1.45	7.44E-01	1.06	1.01	1.92E-01	1.22
Phosphatidylcholine	PC(24:3)	pos	(4:0/20:3)	3.93	1.34	5.25E-01	1.09	1.20	1.43E-01	1.29
	PC(35:0)	pos	(16:0/19:0)	9.96	1.42	7.12E-02	0.51	1.94	1.29E-01	0.60
	PC(38:6)	neg	(18:2/20:4)	7.76	1.40	5.80E-01	1.05	1.15	7.08E-01	1.05
	PC(39:5)	neg	(19:1/20:4)	8.59	1.16	8.35E-01	1.04	1.30	9.58E-01	1.01
	PC(42:10)	neg	(20:4/22:6)	6.94	1.25	8.83E-01	1.02	1.01	3.80E-01	1.22
	PC(42:9)	neg	(22:5/20:4)	7.32	1.02	9.99E-01	1.00	1.20	8.33E-01	1.10
	PC(O-21:0)	pos	(8:0e/13:0)	4.87	1.60	5.48E-01	1.12	1.02	2.16E-01	1.21
	PC(O-38:4)	pos	(18:0e/20:4)	9.22	1.07	2.49E-01	0.75	1.21	7.75E-01	0.95
	PC(P-36:2)	pos	(18:0p/18:2)	8.98	1.15	1.51E-01	0.68	1.70	2.72E-01	0.77
	PC(P-40:7)	pos	(16:0p/24:7)	8.70	1.04	1.84E-01	0.87	1.14	6.38E-01	0.95
Phosphatidylethanolamine	PE(36:5)	neg	(16:0/20:5)	7.56	1.03	8.61E-01	1.09	1.00	4.59E-01	1.65
	PE(40:6)	pos	(18:0/22:6)	8.58	1.35	5.16E-01	1.30	1.06	6.76E-02	2.37
	PE(42:6)	neg	(18:0/24:6)	8.66	1.13	4.48E-01	1.09	1.26	9.74E-01	1.00
Phosphatidylglycerol	PG(P-32:4)	neg	(12:0p/20:4)	2.80	1.04	3.39E-01	0.34	1.32	2.11E-01	0.35
Phosphatidylinositol	PI(37:4)	neg	(17:0/20:4)	7.55	1.28	5.12E-01	1.08	2.03	5.59E-03	1.46
	PI(38:6)	neg	(16:0/22:6)	6.85	1.25	8.68E-01	1.03	1.48	3.58E-03	1.67
Pphingomyelin	SM(d33:1)	neg	(d16:0/17:1)	7.77	1.02	6.45E-01	0.95	1.49	9.16E-01	0.99
	SM(d42:6)	pos	(d16:0/26:6)	7.91	1.72	1.27E-01	4.32	1.28	1.79E-02	6.51
	SM(d44:7)	pos	(d18:1/26:6)	9.06	1.42	1.87E-01	0.57	1.17	4.16E-01	0.54
Triglyceride	TG(52:4)	pos	(16:0/18:2/18:2)	12.19	1.13	4.37E-01	1.22	1.97	6.73E-05	6.74
	TG(54:6)	pos	(16:0/16:1/22:5)	12.00	1.13	4.13E-01	1.26	1.46	1.45E-03	4.88
	TG(54:7)	pos	(16:0/18:2/20:5)	11.78	1.52	9.24E-02	1.68	1.01	2.16E-02	4.18
	TG(55:1)	pos	(16:0/18:1/21:0)	13.40	1.48	2.78E-01	1.43	1.25	2.90E-01	1.43
	TG(55:5)	pos	(17:0/18:1/20:4)	12.55	1.09	8.90E-01	1.05	1.48	8.03E-04	6.06
	TG(55:7)	pos	(15:0/18:2/22:5)	11.87	1.19	4.69E-01	1.24	1.27	4.68E-03	5.36
	TG(55:8)	pos	(15:0/18:2/22:6)	11.70	1.29	2.58E-01	1.35	1.22	1.53E-02	5.42
	TG(56:7)	pos	(18:0/18:2/20:5)	12.14	1.10	3.47E-01	1.24	1.37	5.08E-03	4.54
	TG(56:8)	pos	(16:0/18:2/22:6)	11.90	1.84	2.37E-02	1.97	1.24	1.70E-02	5.08
	TG(57:9)	pos	(17:1/18:2/22:6)	11.73	1.35	3.11E-01	1.29	1.18	1.04E-02	5.19
	TG(58:10)	pos	(18:2/20:4/20:4)	11.64	2.16	4.97E-03	1.76	1.08	2.83E-02	4.86
	TG(58:8)	pos	(18:0/20:4/20:4)	12.31	1.40	2.52E-01	1.26	1.48	5.06E-03	4.83

TG(58:9)	pos	(18:1/18:2/22:6)	11.91	1.86	2.86E-02	1.56	1.30	9.60E-03	5.91
TG(59:5)	pos	(23:1/18:2/18:2)	12.97	1.29	4.16E-01	1.48	1.21	5.82E-02	19.73
TG(59:8)	pos	(19:0/18:2/22:6)	12.39	1.51	2.48E-01	1.45	1.42	1.01E-02	4.39
TG(60:11)	pos	(16:0/22:5/22:6)	11.72	1.43	9.92E-02	1.42	1.22	1.92E-02	4.58
TG(60:8)	pos	(20:1/18:1/22:6)	12.54	1.25	2.16E-01	1.40	1.81	3.76E-03	7.85
TG(60:9)	pos	(18:0/20:4/22:5)	12.30	1.08	6.02E-01	1.13	1.90	6.22E-04	5.29
TG(62:11)	pos	(18:0/22:5/22:6)	12.07	1.25	3.94E-01	1.21	1.50	5.57E-03	5.77
TG(62:12)	pos	(22:4/18:2/22:6)	11.71	1.65	6.19E-02	1.56	1.49	6.37E-03	5.84
TG(62:13)	pos	(18:1/22:6/22:6)	11.61	1.47	5.00E-02	1.67	1.19	2.46E-02	4.49
TG(62:7)	pos	(16:0/22:6/24:1)	13.10	1.11	4.48E-01	1.27	1.25	3.47E-03	11.29

Note: the same with supplementary table 2.

Supplementary Table 3. *Z*-scores of potential disturbed lipid pathways

	ACO	CAL
TG-DG	-0.89	1.01
PG-CL	-0.57	1.73
PC-DG	0.54	-0.72
PC-DG	0.54	-0.72
PS-PE	1.31	1.14
PE-PS	1.49	1.35
PE-PC	1.58	0.22
PC-PS	2.34	2.71
LPC-PC	2.16	0.84
PC-LPC	3.87	1.60
PI-PA	2.82	1.91
LPE-LPC	2.75	3.39
PA-DG	1.56	-0.71
DG-PA	2.09	1.76
PE-LPE	-0.12	2.90
LPE-PE	0.27	3.44
PE-PA	1.30	1.68

Methods

1. The detailed information of MS detection

The MS spray voltages were 3.0 kV and 2.8 kV in the ESI^+ and ESI^- mode, respectively. The capillary temperature was set at 350 ℃ with the sheath gas flow rate at 35 arb, aux gas flow rate at 15 arb and sweep gas flow rate at one arb. The heater temperature was set at 350 ℃. The S-Lens RF level was set at 50. The MS was operated at a resolving power of 70,000 in full-scan mode (scan range: 250-1500 m/z; automatic gain control target: le^6) and of 17,500 in the Top 10 data-dependent MS mode (stepped normalized collision energy: 15, 25 and 35 both in positive and negative mode; injection time: 100ms; isolation window: 1 m/z; automatic gain control target: $2e^5$) with dynamic exclusion setting of 6.0 s.

2. Quality control (QC)

To ensure the quality and repeatability of the data during the whole analysis process, pooled QC samples were prepared by a mixture of all the myocardia or serum samples. Same pretreatment methods and parameters as above-mentioned were used. PCA-X plots of QC indicate that the sample analysis sequence had satisfactory stability and repeatability (Supplementary Fig. 2).
